# Expression Profile of Sorghum Genes and *Cis*-Regulatory Elements under Salt-Stress Conditions

**DOI:** 10.3390/plants11070869

**Published:** 2022-03-24

**Authors:** Solji Lee, Donghyun Jeon, Sehyun Choi, Yuna Kang, Sumin Seo, Soonjae Kwon, Jaeil Lyu, Joonwoo Ahn, Jisu Seo, Changsoo Kim

**Affiliations:** 1 Department of Crop Science, Chungnam National University, Daejeon 34134, Korea; solji2m@o.cnu.ac.kr (S.L.); sehyun@o.cnu.ac.kr (S.C.); dkwl3120@cnu.ac.kr (Y.K.); 2 Department of Smart Agriculture Systems, Chungnam National University, Daejeon 34134, Korea; jemdong@cnu.ac.kr (D.J.); seosumin@cnu.ac.kr (S.S.); 3 Korea Atomic Energy Research Institute (Advanced Radiation Technology Institute), Jeongeup 56212, Korea; soonjaekwon@kaeri.re.kr (S.K.); jaeil@kongju.ac.kr (J.L.); joon@kaeri.re.kr (J.A.); su1545@kaeri.re.kr (J.S.); 4 Department of Horticulture, College of Industrial Sciences, Kongju National University, Yesan 32439, Korea

**Keywords:** salt tolerance, QuantSeq technology, differentially expressed genes, promoter analysis

## Abstract

Salinity stress is one of the most important abiotic stresses that causes great losses in crop production worldwide. Identifying the molecular mechanisms of salt resistance in sorghum will help develop salt-tolerant crops with high yields. Sorghum (*Sorghum bicolor* (L.) Moench) is one of the world’s four major grains and is known as a plant with excellent adaptability to salt stress. Among the various genotypes of sorghum, a Korean cultivar Nampungchal is also highly tolerant to salt. However, little is known about how Nampungchal responds to salt stress. In this study, we measured various physiological parameters, including Na^+^ and K^+^ contents, in leaves grown under saline conditions and investigated the expression patterns of differentially expressed genes (DEGs) using QuantSeq analysis. These DEG analyses revealed that genes up-regulated in a 150 mM NaCl treatment have various functions related to abiotic stresses, such as ERF and DREB. In addition, transcription factors such as ABA, WRKY, MYB, and bZip bind to the CREs region of sorghum and are involved in the regulation of various abiotic stress-responsive transcriptions, including salt stress. These findings may deepen our understanding of the mechanisms of salt tolerance in sorghum and other crops.

## 1. Introduction

Abiotic stresses, such as high temperatures, droughts, floods, salinity, and other natural disasters, limit plant growth and development, resulting in significant yield losses. Of these, soil salinization is one of the strongest abiotic stresses and drastically limits crop production. Contrary to the current increasing population, the land used for agriculture is gradually decreasing every year [1]. According to the FAO, over 6% of the world’s arable land is being affected by salinity [2]. Soil salinity is increasing by 10% every year due to low precipitation, weathering of native rocks, and saline irrigation [3]. Excessive salinity in soil has high electrical conductivity, low water potential, and induces ionic stress, impairing biological processes such as cellular ionic homeostasis, membrane stability and photosynthesis in plants [4,5]. Additionally, due to insufficient absorption of nutrients, it causes excessive production of toxic ions such as reactive oxygen species (ROS), reducing crop yield and quality [6,7,8]. In recent years, great interest has been raised about the role and action of plant nutrients in plant abiotic stress tolerance [9]. In general, an increase in soil NaCl concentration has been reported to induce an increase in the Na and Cl contents and a decrease in the N, K, Ca, P, and Mg contents in plants. It is important to monitor N, K, P, and Zn because the action of these nutrients can limit plant development [10,11]. Therefore, to produce crops with high quality and yield under salt stress, it is important to identify the mechanisms of the salt response and discover genes that are expressed differently under salt stress conditions [12].

Although the ultimate goal of plant stress studies is to develop resistant/tolerant varieties, the resistance/tolerance mechanism still remains a complex problem with varying responses at the molecular, metabolic, and physiological levels. To understand the salt tolerance mechanisms of plants, a series of responses to salt stress is desired. Functional analysis of salt-related genes provides information to enrich signaling networks and tolerance to salt stress.

Gene expression regulation for salt stress conditions includes transcriptional regulation and RNA processing. In recent years, RNAseq has been widely used as a method to investigate the expression of genes in response to salt stress [13,14,15,16]. RNAseq technology can reveal genes and molecular pathways that are differentially expressed across multiple biological conditions [17].

While RNAseq is generally considered unbiased, some biases may be introduced due to fragmentation and library construction steps. To minimize this bias, 3′-RNA sequencing methods such as TagSeq and QuantSeq have recently been developed as a more cost-effective method for quantifying gene expression levels [18]. The QuantSeq method sequences only the 3′-end of the RNA fragments which are mostly 3′-untranslated regions (3′-UTR), enabling the quantification of gene expression with fewer reads per sample than standard RNAseq. Consequently, QuantSeq can efficiently reduce the required sequencing depth and data processing time per sample [19,20].

Research on *cis*-regulatory elements has been enriched with recent advances in next generation sequencing (NGS) technology. Regulation of gene transcription in plants is driven by a number of transcription factors [21]. *Cis*-regulatory elements (CREs) are important components of genetic regulatory networks and are DNA sequences in non-coding regions. Typically, transcription factors bind to the region of CREs to regulate the transcription and expression of neighboring genes [22,23]. Thus, identifying the function of plant CREs has an essential role in determining the tissue-specific or abiotic stress response expression patterns of the target gene [24].

Sorghum (*Sorghum bicolor* [25] Moench) is a food crop originating from Africa and is widely cultivated for animal feed and biomass production in tropical and subtropical regions [26]. It is a diploid with 10 basal chromosomes and has a small genome size (~730 Mbp) [27,28]. In addition, it is a multi-purpose crop belonging to the PACMAD clade of the Poaceae family, a C4 plant characterized by high photosynthetic ability and productivity and has excellent adaptability to stresses such as moderate salinity and drought [27,29,30]. As such, sorghum, possessing a small genome size and adaptability to various environments and stresses, is very valuable as a model cereal for comparative, structural, and functional genomic studies to improve agriculturally important traits. Sorghum is the fourth most important crop in the world after rice, wheat and maize [30]. Although sorghum is an agriculturally important crop, less is known about its mechanisms or responses to survival in salt stress environments compared to other crops. There are significant genotypic differences in the response of sorghum cultivars to salt [31]. Among various sorghum varieties, Nampungchal (chal means ‘sticky’ in Korean) is a salt-tolerant sorghum that exhibits excellent yield characteristics in reclaimed land [32]. Nampungchal was bred by the National Institute of Food Science and Technology in Korea to cultivate sorghum with lodging resistance and abiotic stress tolerance. After collecting sorghum ‘Namhae’, a native species from Namhae city, Gyeongsangnam-do, Korea in 2009, it was finally bred through a yield and regional adaptation test from 2011 to 2012 through pedigree breeding. Despite its tolerance to salt stress, there is no ongoing research regarding the adaptation of Nampungchal to salt stress.

The purpose of this study was to investigate the expression difference of the salt stress-related genes from a sorghum cultivar, Nampungchal, under various salt stress conditions. In addition, promoter analysis was performed to search for putative *cis*-regulatory elements associated with salt tolerance. After salt stress treatment, various physiological parameters of Nampungchal were evaluated, and differentially expressed genes were identified through QuantSeq.

## 2. Results

When the leaves of Nampungchal grew from three to four leaves, the salt stress treatment was started, and the duration was measured. Unlike seedlings grown under normal conditions, we clearly observed reduced growth in both the leaves and roots under severe salt stress conditions (150 mM NaCl). The reduced growth may be caused by a variety of different physiological factors; in turn, those physiological factors were basically governed by changes in specific gene expression levels. Consequently, we investigated some physiological and gene expression changes presumably affected by salt stress conditions in plants.

### 2.1. Effects of Physiological Factors under Salt Stress

**Figure 1:**
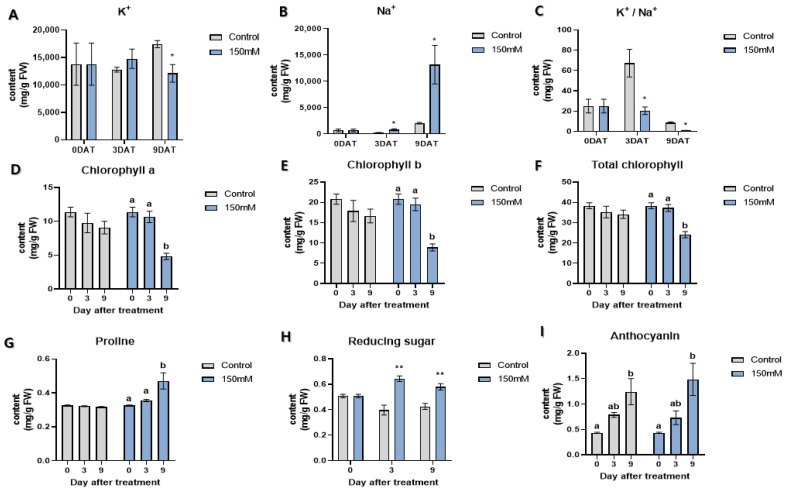
Physiological parameters under salt stress conditions in the leaves of Nampungchal cultivar. (**A**) Potassium content, (**B**) sodium content, (**C**) K^+^/Na^+^ content, (**D**) chlrophyll a content, (**E**) chlorophyll b content, (**F**) total chlorophyll content, (**G**) proline content, (**H**) reducing sugar content, (**I**) anthocyanin content. a, b: Different letters indicate significant differences among different days (ANOVA followed by Tukey’s test at *p* < 0.05). * means *p*-value = 0.05, ** means *p*-value = 0.1.

Significant changes in the Na^+^ and K^+^ contents were observed in the leaves when compared with plants grown under normal conditions after the treatment with 150 mM NaCl for 3 and 9 days. The Na^+^ content tended to increase significantly at both the 3 days after treatments (DAT) and 9 DAT, especially at the 9 DAT. Compared to the control group, the K^+^ concentration increased slightly in the 3 DAT but showed a tendency to decrease significantly in the 9 DAT. Consequently, K^+^/Na^+^ decreased significantly in both the 3 and 9 DAT after the salt stress treatment (Figure 1A–C).

The changes in the chlorophyll contents of Nampungchal under salt stress showed a general trend. Both chlorophyll a and b and total chlorophyll showed a tendency to decrease after the salt stress treatment in both the 3 and 9 DAT, but only the 9 DAT showed a significant decrease (Figure 1D–F).

Proline can act as an osmoprotectant to various abiotic stresses in plants and is considered as a parameter for evaluating the intensity of stress levels. Compared to normal conditions, the levels of the proline contents were significantly elevated under the 150 mM NaCl treatment at 9 DAT. After the 150 mM NaCl treatment, the proline content tended to increase by 48% at 9 DAT compared to the control group and increased significantly by 35% at 9 DAT compared to the 3 DAT (Figure 1G).

Reducing sugars can also act as an osmoprotectant in plants, reflecting the intensity of osmotic stress levels caused by drought or salt in many plants. Three and 9 DAT for salt stress showed significant changes in the reducing sugar content. Compared to the control group, the 3 and 9 DAT showed a high tendency of 62% and 35%, respectively (Figure 1H).

Anthocyanin is not a parameter to determine abiotic stress levels, but it can be induced by plants under osmotic stresses due to its anti-oxidative activities. The anthocyanin content tended to increase with the longer salt stress treatment period and higher concentrations of salt. However, like the salt-treated leaves, the control group also showed a tendency to increase the anthocyanin content, which was not statistically different from each other (Figure 1I).

### 2.2. QuantSeq Data Generation and Assembly

Samples for QuantSeq analysis were obtained from two replicate plants from leaves and roots of the 0 and 150 mM NaCl treatments at 3 DAT. These samples were sequenced using the Illumina NextSeq 500 platform, then data filtering was performed to remove low quality reads and adapter sequences. Leaves and roots grown on 3 days under normal conditions (two technical replications) obtained 15,679,862, 16,957,637, 17,039,504, and 16,990,159 reads, respectively. The leaves and roots of the seedlings at 3 days after the salt stress treatment (two technical replications) obtained 15,882,789, 19,354,751, 17,225,187, and 19,132,885 reads, respectively. The reads of each sample showed more than 93% mapping rate to BTx623, the sorghum reference genome.

### 2.3. Identification of DEGs in Response to Salt Stress

**Figure 2:**
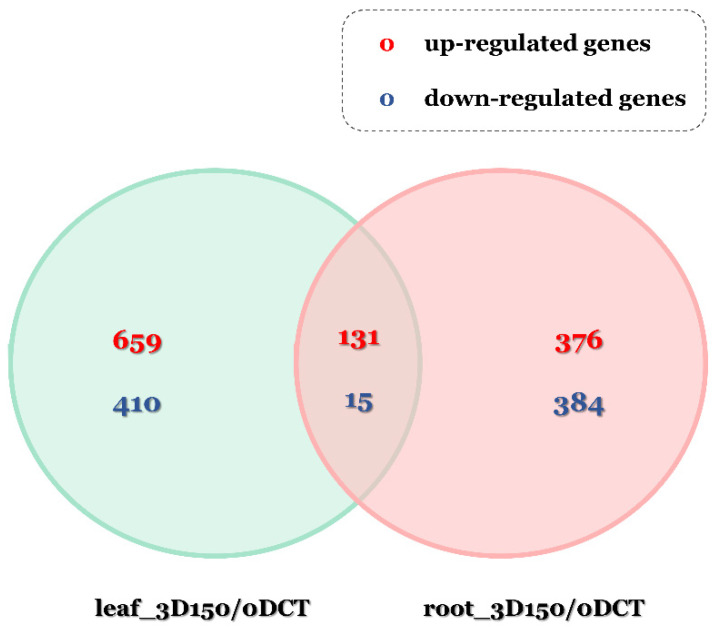
Statistics of differentially expressed genes under salt stress conditions and normal growth conditions (3D150; 150 mM NaCl treatment at 3 DAT, 3DCT; 0 mM NaCl treatment at 3 DAT).

A fold change value of gene expression ≥4.00 and normalized data (log_2_) ≥ 4.00 and *p*-value ≤ 0.05 were used to identify differentially expressed genes. As a result, more DEGs were present in the leaves than in the roots, and both the leaves and roots had more up-regulated genes than down-regulated ones. A total of 1215 genes were differentially expressed when compared between the control and 150 mM NaCl treatment in the leaves at 3 DAT, of which 790 and 425 genes were up-regulated and down-regulated, respectively. When compared to the roots grown under normal conditions and salt stress (150 mM NaCl), a total of 906 genes differentially expressed were found. Of these, up-regulated and down-regulated genes were 507 and 399, respectively. A total of 146 genes were differentially expressed both in the leaves and roots, of which 131 genes were up-regulated and 15 genes were shown to be down-regulated (Figure 2). The commonly up-regulated genes had a variety of functions, among which there were many genes that function as ethylene-responsive transcription factor (ERF), dehydration-responsive element-binding protein, zinc finger protein, and a probable WRKY transcription factor, well-known to be abiotic stress-responsive genes (Table 1). Among the down-regulated genes, functions such as probable polyamine oxidase were shown to be representative (Table 2).

### 2.4. Functional Classification and KEGG Enrichment Analysis of Salt-Responsive DEGs

**Figure 3:**
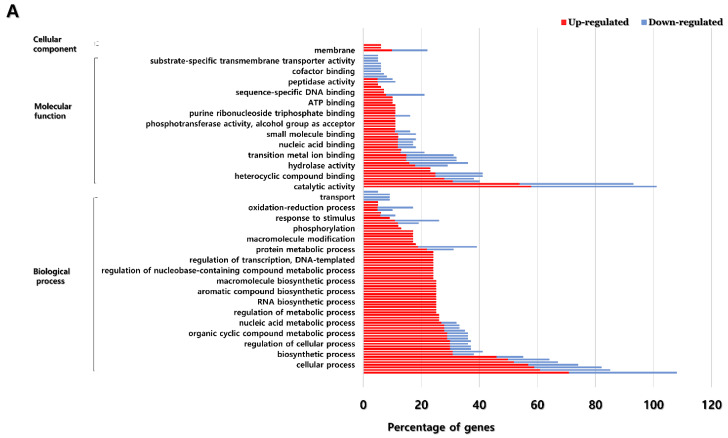

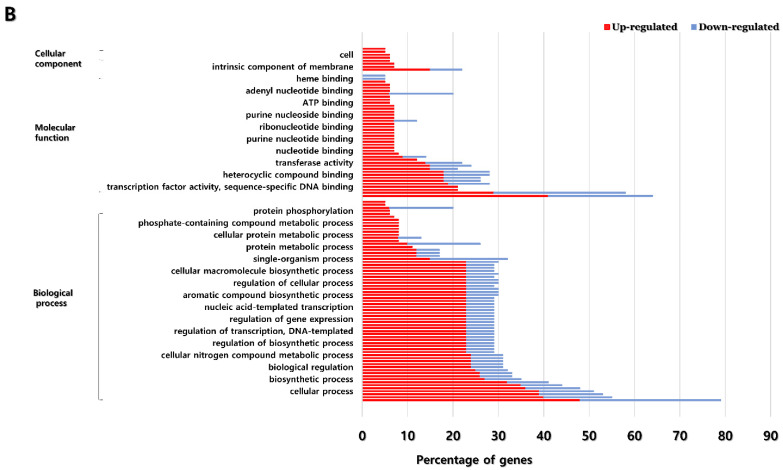
GO enrichment analysis for DEGs in leaves (**A**) and root (**B**).

GO enrichment analysis of differentially expressed genes (fold change ≥4, normalized data ≥4, *p*-value ≤ 0.05) between treated (3 days after 150 mM NaCl treatment) and control plants was submitted to singular enrichment analysis (SEA) using AgriGo 2.0 (http://systemsbiology.cau.edu.cn/agriGOv2, accessed on 10 September 2020). The functional classification of DEGs was divided into three categories: biological process, molecular function, and cellular component. In the case of biological processes, most genes in the metabolic process (GO: 008152), organic substance metabolic process (GO: 0071704), and primary metabolic process (GO: 0044238) in both the leaves and roots were upregulated and downregulated. Molecular function showed the greatest enrichment of genes up and down regulated in categories related to catalytic activity (GO: 0003824) and binding (GO: 0005488). For cellular components in the leaves, the upregulated genes were significantly abundant in the membrane (GO: 0016020) and cell part (GO: 0032990) categories, and the downregulated genes were most abundant in the membrane (GO: 0016020) category. In the case of the cellular component in the root, the upregulated genes were most representative in the membrane (GO: 0016020) and the intrinsic component of membrane (GO: 0031224) categories, and the downregulated genes were most abundant in the membrane (GO: 0016020) category. There were many GO terms associated with abiotic stress in leaves: nitrogen compound metabolic process (GO: 0006807), cellular nitrogen compound metabolic process (GO: 0034641), cellular aromatic compound metabolic process (GO: 0006725), aromatic compound biosynthetic process (GO: 0019438), response to stimulus (GO: 0050896), proteolysis (GO: 0006508), and response to stress (GO: 0006950). Of these, only up-regulated genes existed in the response to stimulus and response to stress categories (Figure 3).

**Figure 4:**
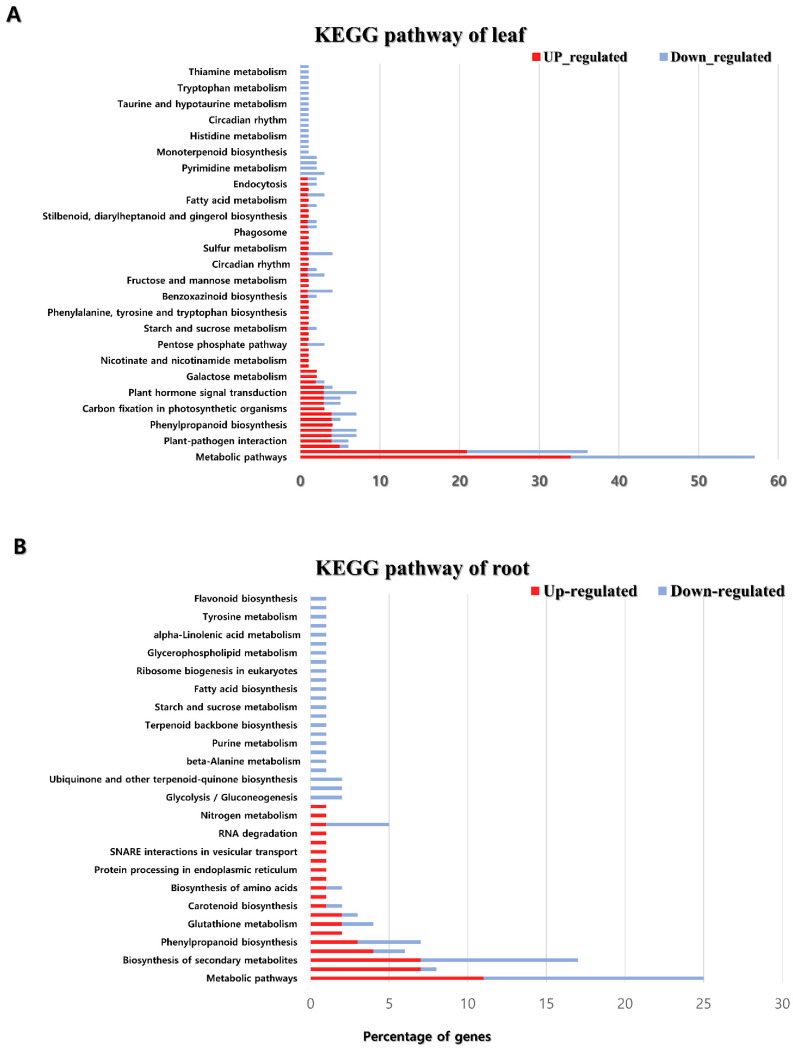
KEGG pathway analysis of leaf (**A**) and root (**B**) DEG.

The KEGG pathway was analyzed to identify the biological pathways of genes differentially expressed between plants grown under normal and salt treatment conditions. Most of the genes up-regulated in the leaves were found to be most abundant in the metabolic pathways, biosynthesis of secondary metabolites, and biosynthesis of amino acids pathways. In addition, genes that were down-regulated in the leaves have been shown in metabolic pathways, biosynthesis of secondary metabolites pathway, and plant hormone signal transduction pathway (Figure 4). The metabolic pathway showed the most up-regulated genes in the roots, followed by plant–pathogen interaction, biosynthesis of secondary metabolites, and the MAPK signaling pathway. Down-regulated genes in the roots were abundant in the order of metabolic pathways, biosynthesis of secondary metabolites, phenylpropanoid biosynthesis, and plant hormone signal transduction pathway (Figure 4).

### 2.5. Identification of Potential CRE Functions Associated with Abiotic Stress and Exploration of Related Sorghum Genes

Currently, the whole genome of Nampungchal has not been sequenced. Therefore, to search for *cis*-regulatory elements related to abiotic stress, 1 kb up-stream regions from the sorghum reference genome BTx623 were extracted, and the analysis was performed using the New PLACE database, which has a database of *cis*-regulatory elements of plants. A total of 10,189 *cis*-regulatory elements were found, of which only 3757 were left by filtering out only the data with the highest e-values and similarity ≥90% based on the BlastN search. After the duplicate values were removed, a final 179 *cis*-regulatory elements were obtained. The function related to each *cis*-regulatory element was referenced from the New PLACE site.

The list of genes and functions of *cis*-regulatory elements related to abiotic stress is summarized in Table 3. About 35 of the 179 *cis*-regulatory elements have been shown to have functions related to abiotic stress. These functions include MYB, WRKY, and bZIP, one of the most well-known transcription factors related to plant abiotic stress, as well as ABA, stress, and dehydration responsive elements. In addition, among these *cis*-regulatory elements, many elements related to the ABA reaction were shown.

**Figure 5:**
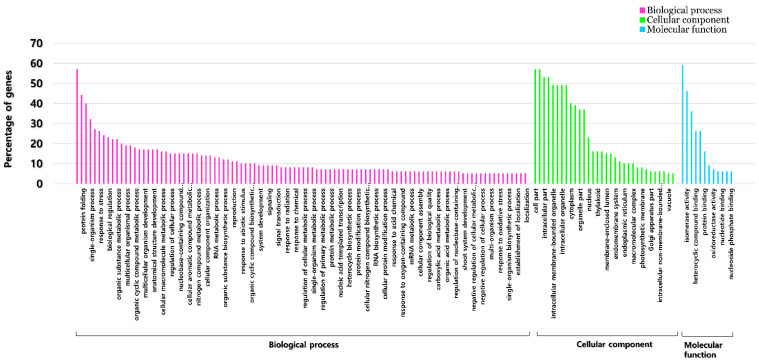
GO analysis of ‘Nampungchal’ genes related to *cis*-regulatory element.

A total of 80 Nampungchal genes that bind to the *cis*-regulatory elements were confirmed to match the GO categories of those genes. The data before the analysis were given the following options: the Fisher method for the statistical test, the Yekutieli (FDR under dependency) for the multi test adjustment, and a significance level of 0.05. Those 80 genes were matched to the categories of biological process, cellular component, and molecular function. Among them, there were many categories related to stress: response to stress, response to abiotic stimulus, response to radiation, cellular response to stimulus, cellular response to stress, and response to oxidative stress (Figure 5).

Of the 80 Nampungchal genes that matched the putative *cis*-regulatory elements found in this study, 55 genes and functions related to abiotic stress were found (Table 4). The genes functioned in many orders, such as CYP (cyclophilin), ABA (abscisic acid), FKBP (FK506-binding proteins), and 9-*cis*-expoxycarotenoid dioxygenase.

### 2.6. Validation of DEG Profiles by qRT-PCR

**Figure 6:**
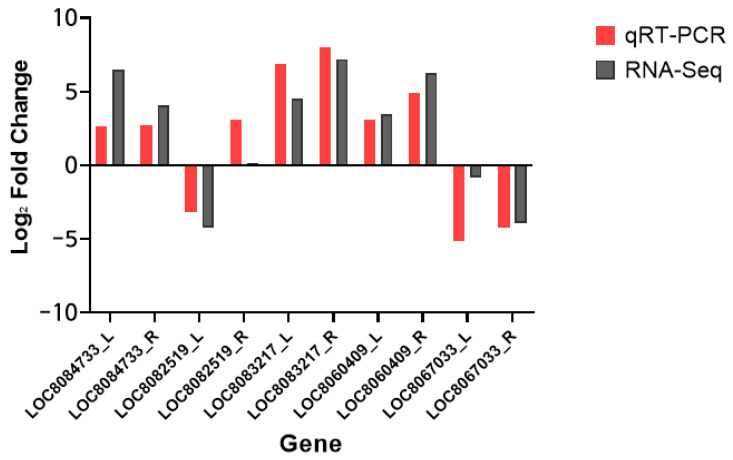
Validation of five DEGs using qRT-PCR. The pink bars correspond to qRT-PCR and the gray bars correspond to RNA-Seq. The *x*-axis represents genes and the *y*-axis represents log_2_ fold change. L and R after the gene names represent leaves and roots, respectively.

Based on the QuantSeq analysis, five differentially expressed genes LOC8084733, LOC8082519, LOC8083217, LOC8060409, and LOC8067033 were randomly selected for qRT-PCR analysis for the validation of DEG analyses. Using the designed primers, expression profiles of the five target genes were tested by qRT-PCR (Figure 6). The results showed that the expression trends of the LOC8084733, LOC8082519, LOC8083217, LOC8060409, and LOC8067033 genes through qRT-PCR and QuantSeq showed consistent patterns. These results show that the transcriptome data reflect the response of Nampungchal leaves and roots to salt stress.

## 3. Discussion

Salinity is one of the most harmful abiotic stresses that inhibit plant growth and development. Most studies investigating the response of plants to salt stress have looked at the stress response through the leaves and root organs of plants. The response of plants to high concentrations of salt in the soil first induces osmotic stress to inhibit root moisture absorption [33] and induces ionic stress to impair photosynthesis including the disruption of chlorophylls, resulting in decreased growth or death of the leaf and root tissues [34]. Therefore, we first observed physiological changes mostly in the leaves of Nampungchal and performed transcriptome analysis from both the leaves and roots to predict how sorghum responds to the high level of salt stress.

Plants, to survive saline conditions, activate defense mechanisms through a variety of strategies and exhibit several physiological and metabolic changes. These reactions can be considered as biological indicators of changes in salt response, which are mostly triggered by the changes of gene expression. Previous physiological studies of various crop species, such as maize (*Zea mays* L.), rice (*Oryza sativa* L.), barley (*Hordeum vulgare* L.), and cotton (*Gossypium davidsonii*), have observed changes in the physiological response of plants to salinity and even predicted changes in gene expression [34,35,36,37]. Ionic homeostasis such as Na^+^ and K^+^ in plant cells has an important role in salt tolerance and is essential for growth. Maintaining the optimal K^+^/Na^+^ ratio within the cell to survive the high salt concentration of the soil has an important role in salt tolerance [38,39]. After the 150 mM salt stress treatment in Nampungchal, the level of Na^+^ in the leaves noticeably increased compared to the normal condition. It also dramatically increased as the treatment period was prolonged from 3 to 9 days. K^+^ is also an essential component for salt tolerance [40]. In the current study, despite the sudden increase in Na^+^ at 9 DAT, it was observed that the level of Na^+^ did not dramatically increase until 3 DAT, presumably indicating that this sorghum cultivar may have the avoiding mechanisms of salt-absorption where a plant can buffer against an excessive amount of salt [41]. It was also observed that the ratio of K^+^ to Na^+^ was maintained throughout the treatment, consistent with the fact that proper maintenance of K^+^/Na^+^ under salt conditions has an important role in plant salt tolerance [42]. Considering that Nampungchal has a moderate level of salt tolerance, it may be expected that the fluctuation of the ratio would be less than other salt-susceptible sorghum cultivars. This may need to be checked in our future salt stress studies by adding some salt-susceptible cultivars.

In general, plants under salt stress gradually lose their ability to absorb water. Due to this osmotic stress, plants use many strategies to conserve water contents by stomatal closure which in turn reduces the transpiration rate and inhibits the absorption of CO_2_, ultimately resulting in decreased growth by limiting photosynthesis [34,43,44]. For example, chlorophyll a and b content decreased after NaCl treatment in the leaves of rice and beans [45,46]. We also evaluated the chlorophyll a/b and total chlorophyll contents to determine the photosynthetic pigments against salt stress in Nampungchal. The contents of chlorophyll a/b and the total chlorophyll in the leaves of 3 DAT showed a slight increase compared to the control group, but showed a significant decrease in 9 DAT, indicating that Nampungchal was not significantly affected by salt in the early stages of growth due to its basic ability to tolerate salt stress in the early stages. This was consistent with the result of the potassium ion contents previously discussed.

Maintaining the balance of osmotic pressure, which induces a continuous inflow of water against changes in external osmotic pressure, or reduces outflow, is known to be a function of osmolytes. Solutes such as proline, sugars, sugar alcohols, and polyols can function as osmolytes [4,47]; consequently, these contents are good indicators to see if plants are affected by osmotic stresses. Of these, the accumulation of proline is one of the adaptation processes of plants for osmotic regulation under various abiotic stresses, including salt [48]. Many studies have reported that proline accumulation is concordant to the damaging response of a plant to salt stress [49,50]. We showed the same trend that the proline content in the leaves gradually increased after the treatment of 150 mM NaCl, which can be interpreted as a damaging response to salt stress.

In general, plants exposed to salt stress accumulate reducing sugars and sucrose to resist stress and increase osmotic function, as previously stated. Therefore, the sugar content can be used as a physiological indicator of salt resistance evaluation as well [51,52,53]. In the current study, the change in the reducing sugar contents in the salt-treated plants significantly increased both at 3 and 9 DAT compared to the control plants, supporting the results of previous studies.

We recently published a paper about the physiological response after salt stress treatment in a salt-sensitive sorghum variety [54]. In this paper, the change in proline contents after salt stress treatment in Sodamchal (a salt-sensitive sorghum variety) tented to gradually increase over time, and the reducing sugar contents were to decrease significantly, showing the opposite trends to Nampungchal. Based on these results, proline contents could be a key indicator to determine the intensity of salt-tolerance in sorghum. On the other hand, in the case of reducing sugar contents, Sodamchal showed the opposite trend to Nampungchal, indicating that the accumulation of reducing sugar contents would help increase the tolerance to salt in plants.

An increasing anthocyanin content is known as a mechanism for protecting plants and reproductive tissues against saline conditions and various abiotic stresses [55]. It is also a secondary metabolite that can be induced by oxidative stress possibly caused under salt or drought conditions and has the potential to increase salt tolerance by protecting cells from oxidative damage [56,57]. The accumulation of anthocyanin content, which can help with this salt tolerance, was also observed in the leaves of Nampungchal under salt conditions. However, the increasing patterns in the treated plants were not significantly different from those in the control plants, indirectly indicating that the contents of anthocyanin may not be a key factor in protecting Nampungchal from salt stress.

The results of these physiological responses enabled us to explore and predict changes in differentially expressed genes for salt stress. In this study, differentially expressed genes were found by comparing leaves and roots under normal and salt conditions. Totals of 1215 and 906 up- and down-regulated differentially expressed genes were found in the leaves and roots, respectively, and each gene had various functions related to salt stress. Among the functions of DEGs that are commonly up-regulated in leaves and roots, ethylene responsive transcription factor (ERF) and dehydration-responsive element-binding protein (DREB) were typically associated with salt stress.

ERF, belonging to the APETALA2/ERF family, is an important plant-specific transcription factor in the defense against biotic and abiotic stress responses in many plants [58,59]. When plants are in an adverse environment, the ERF protein binds to the GCC and DRE/CRT boxes in the promoter regions to activate or inhibit stress-related genes [60,61]. Many studies have been conducted from the past to the present showing that ERF overexpression increases plant tolerance to NaCl. In *Arabidopsis*, overexpression of ERF96 was induced by NaCl treatment, showing improved salt tolerance in terms of seed germination and seedling growth [62]. Overexpression of *TERF1* enhances resistance to stress by regulating the expression of genes that respond to drought and salt in rice [63]. In our study, various ERF proteins were represented in the up-regulated genes under salt stress conditions, indicating that ERF has an important role in sorghum as well. The DREB protein is involved in plant stress signaling pathways and known as an important transcription factor conferring tolerance to a variety of stresses [64]. They also have a key role in pathways associated with ABA-independent stress tolerance, and one of their subfamily, DREB2, induces stress-responsive gene expression by drought and high salt concentrations [65]. Some studies have shown that the expression of the DREB gene increases resistance to abiotic stress. DREB, one of the transcription factors that is transcriptionally up-regulated due to lack of water, was overexpressed in soybean, and then physiological and transcriptome analysis was performed. As a result, proline accumulation was improved and salt tolerance was increased [66,67]. Many of these DREB-functioning genes were up-regulated in Nampungchal under saline conditions, assuming that DREB is also a controlling factor to improve salt-tolerance in sorghum based on our transcriptomic studies.

While there are genes that make them resistant to salts by helping them defend against salt conditions, the expression of a few genes may act as negative regulators under saline stress. For example, PAO is a key enzyme that induces polyamine catabolism and induces the production of ROS like H_2_O_2_. H_2_O_2_ has a different role in response to stress at low and high concentrations. They induce the expression of stress-responsive genes to adapt to environmental stress at low concentrations. However, at high concentrations, it causes oxidative stress, resulting in PCD (programmed cell death), which eventually inhibits growth [68,69]. These PAO-functional genes were down-regulated in the Nampungchal leaves and roots under salt conditions, so they did not appear to work in the salt-tolerant sorghum cultivar.

We additionally identified differences between a salt-sensitive cultivar (Sodamchal) and a salt-tolerant cultivar (Nampungchal) by comparing transcriptome analysis data (personal communication with the authors of [54]). After treatment with 150 mM NaCl for 3 days in Nampungchal and Sodamchal, a total of four genes in leaves and roots were up-regulated in Nampungchal and down-regulated in Sodamchal (LOC8065112, LOC8071958, LOC8054678, LOC110433584). Among the genes identified in the leaf, LOC8065112 had a pentatricopeptide repeat-containing protein (PPR) function and LOC8071958 had a probable FBD-associated F-box protein function. These two proteins are reported to regulate plant responses to abiotic stresses and to play essential roles in plant growth and development processes. A previous study reported that overexpressed lines of the PPR protein, SOAR1, have a tolerance to abiotic stress in *Arabidopsis thaliana* [70]. According to a recent study, overexpression of *ATPP2-B11*, an F-box protein, also enhanced salt tolerance in *Arabidopsis thaliana* [71]. Since these genes have opposite expression direction in the two genotypes, these genes may be candidate genes that bring a difference in salt tolerance in sorghum. However, since the function of the two genes identified in the root has not been clearly identified as being associated with salt tolerance, it seems that further research is needed related to salt stress.

Molecular genetic analysis over the last 20 years has found that transcriptome engineering is a promising option to increase the tolerance of abiotic stress in plants [72]. Research on the promoters that regulate gene expression (at the transcriptional level) helps us understand the transcriptional regulations of many genes. The promoters present upstream of the gene coding regions include *cis*-regulatory elements, enhancers, silencers, and insulators, which are specific binding regions for proteins that regulate gene transcription [73,74]. Transcription factors are proteins that regulate the transcription after binding to regions of specific *cis*-regulatory elements residing mostly in the upstream of target genes [75]. Various transcription factors attach upstream of the transcription initiation site to form a transcription initiation complex. After this, RNA polymerase is activated, and transcription of certain stress-responsive genes begins. Through this process, *cis*-regulatory elements provide molecular connections between various metabolic pathways [76]. Knowing the relationship between the structure and function of *cis*-regulatory elements is essential for comparative genomic studies. Recent advances in high-throughput sequencing technologies have enabled the identification of genome-wide potential *cis*-regulatory elements in a variety of crops. We performed an analysis of putative *cis*-regulatory elements to determine which promoters of Nampungchal are associated with salt stress for the prediction of regulatory changes. However, because the whole genome sequence of Nampungchal has not yet been available, *cis*-regulatory elements were found using 1 kb upstream of BTx623 publicly available as the sorghum reference genome. The reason why BTx623 could be used for finding *cis*-regulatory elements in this study is that the functions and sequences of *cis*-regulatory elements are well-conserved among species even when speciation or evolution occurs [77].

As a result of our *cis*-regulatory elements analysis, 35 out of 179 CREs were related to abiotic stress, and these were confirmed to have functions such as MYB, WRKY, and BZIP transcription factors, and ABA. ABA was the most abundant function associated with osmotic stresses. Drought and salinity stress lead to osmotic pressure imbalances, and in turn, plants under osmotic stress accumulate abscisic acid (ABA), a key hormone in stress signaling [78]. These ABAs are essential for plant growth processes, including embryonic development and seed maturation [79]. Various studies have reported that the accumulation of ABA increases the salt tolerance of plants. Treatment with ABA in rice during salt stress conditions improves salt tolerance compared to normal conditions [80]. ABA improves osmotic stress through increased changes in proline and better osmotic control in wheat seedlings [81]. Representative functions in our analysis were ABA and 9-*cis*-epoxycarotenoid dioxygenase. The 9-*cis*-epoxycarotenoid dioxygenase (NCED) is a key enzyme in ABA biosynthesis [82] and is considered a key enzyme that controls stress tolerance [83]. Overexpression of NCED in transgenic tobacco plants increased ABA accumulation and enhanced salt tolerance [84]. OsNCED3 overexpression showed survival rates of 64.9% and 81.6% in transgenic rice under salt conditions, and the survival rate of the wild type was only 54.1%. Therefore, NCED was resistant to salt stress [85]. These results suggest that ABA and NCED have agricultural potential to improve the salt stress tolerance of sorghum as well.

## 4. Materials and Methods

### 4.1. Plant Growth Conditions

In this study, seeds of ‘Nampungchal’ were used as experimental materials. The seeds were obtained from the Rural Development Administration (Jeonju, Korea). Nampungchal is considered to be tolerant to salt stress [32]. The sterilized Nampungchal seeds were sown in plastic pots filled with bed-soil and grown to the seeding stage (in a greenhouse at Chungnam National University, Daejeon, Korea). When the seedlings had grown to about three leaves, the plants were transferred to new pots filled with sterilized sands, and then, they were put in a tray with half-strength Hoagland’s solution so that capillary reaction kept supplying nutrient solutions. This process lasted for about a week to adapt the plants to a nutrient-supplying sandy cultural system. Salt stress conditions were prepared by adding 150 mM NaCl (severe salt stress condition) to half-strength Hoagland’s solution, and normal conditions without salt stress (control conditions) were prepared by adding only half-strength Hoagland’s solution. Each treatment was carried out in three biological replications. Samples were collected from leaves and roots of plants on days 0, 3, and 9 after salt stress treatment. After sampling, all samples were immediately frozen in liquid nitrogen and stored at −80 °C until use.

### 4.2. Physiological Analysis

#### Measurement of the Chlorophyll Contents

Total chlorophyll was extracted from leaves using the method described in [86]. To determine the chlorophyll content, 300 mg of fresh leaves were ground with liquid nitrogen using a mortar. Then, 5 mL of 80% acetone was added, and the mixture was shaken for 15–30 min using a HulaMixer in a dark place without light. The mixture was spun down at 4000 rpm for 30 min at 4 °C. The supernatant was measured for absorbance at 663 nm and 648 nm using spectrophotometry. Chlorophyll contents were calculated as follows:

Chlorophyll a (mg/g) = [12.7 × A663 − 2.69 × A645] × V/1000 × W

Chlorophyll b (mg/g) = [22.9 × A645 − 4.86 × A663] × V/1000 × W

Total chlorophyll (mg/g) = [8.02 × A663 + 20.20 × A645] × V/1000 × W

where V = volume of the extract (mL); W = Weight of fresh leaves (g).

1. Measurement of the K^+^ and Na^+^ in the leaves

Oven-dried fresh leaves were ground into a fine powder. Then, 20 mL of sulfuric acid (conc. H_2_SO_4_) was added to the ground 800 mg sample and mixed for 12 h. Using a heating block (MHB-S Series, Ctrl-M Scientific Co., Cerritos, CA, USA), the temperature was set at 130 °C for 30 min and at 130–150 °C for 2 h, and then slowly heated until the red gas disappeared. After the red gas disappeared, it was digested at 180~200 °C for 1 h. When the solution became black, nitric acid was added. This process continued until the solution became clear. After that, 30 mL of distilled water was added and heated for about 3 min, and then the solution was cooled. Finally, the solution was filtered through a volumetric flask. The filtered solution was analyzed by ICP-AES (Inductively Coupled Plasma-Atomic Emission Spectroscopy, iCAP 7400 Duo MFC, Thermo, Waltham, MA, USA).

2. Measurement of the proline contents in the leaves

The proline contents were determined using the method described by [25]. First, the solution was extracted by mixing 50 mg of fresh leaves and 1 mL of ethanol:water (40:60 *v*/*v*). The reaction mixture was prepared by adding 1% ninhydrin (*w*/*v*) in acetic acid 60% (*v*/*v*) and ethanol 20% (*v*/*v*). Then, a total of 500 µL of the ethanol extract was added to the 1000 µL reaction mixture, which was heated in a 95 °C heating block for 20 min. After that, the absorbance was measured at 520 nm using spectrophotometry. The proline content was calculated using a proline standard curve based on a linear regression model.

3. Measurement of the reducing sugar contents in the leaves

The reducing sugar content was determined based on [87]. After grinding the prepared sample with liquid nitrogen, 7 mL of distilled water and 2 mL of DNS reagent were added to a 300 mg sample. The mixture was heated in boiling water for 5 min and immediately put in ice-water prepared in advance to cool it down for 10 min. The reducing sugar contents were measured by absorbance at 570 nm and calculated using a glucose standard curve based on a linear regression model.

4. Measurement of the anthocyanin level in the leaves

Prepared seedlings were ground in liquid nitrogen using a mortar and pestle. Then, a total of 1.5 mL of extraction buffer was added to a 300 mg ground sample. The mixture was centrifuged at 12,000× *g* for 5 min at room temperature, and the supernatant was transferred to a new tube. The tube was centrifuged once again at 12,000× *g* for 5 min at room temperature. The anthocyanin contents were measured at 530 and 637 nm using spectrometry. The anthocyanin contents (mg/g) were calculated as follows [88]:

[Abs_530_ − (0.25 × Abs_657_)] × 5

### 4.3. Statistical Analysis

Data were presented as the mean of three replicates with SEM error bars and subjected to a one-way analysis of variance (ANOVA) and Duncan’s multiple-range tests (*p* ≤ 0.05 were considered as significant) using IBM SPSS Statistics version 26 software (IBM SPSS, Inc., Chicago, IL, USA).

### 4.4. RNA Extraction and Library Construction for QuantSeq

RNA was extracted from the leaves and roots of Nampungchal on the 3rd day in normal conditions (control, 0 mM NaCl) and after the 150 mM NaCl treatment using the TRIZOL reagent (Invitrogen, Carlsbad, CA, USA). All samples had two biological replicates. The extracted RNA was dissolved in DEPC-treated water, quantified using a Nanodrop ND-2000 spectrophotometer (Thermo Inc., Wilmington, DE, USA), and the quality was checked using Bioanaylzer 2100 (Agilent). Library construction of RNA was performed using the QuantSeq 3′-mRNA-Seq Library Prep Kit (Lexogen, Inc., Wien, Austria) according to the manufacturer’s instructions. Briefly, 500 ng of total RNA was prepared, and an oligo-dT primer containing an Illumina-compatible sequence at its 5′ end was hybridized to the RNA, and reverse transcription was performed. After degradation of the RNA template, second strand synthesis was initiated by a random primer containing an Illumina-compatible linker sequence at its 5′ end. The double-stranded library was purified by using magnetic beads to remove all reaction components. The library was amplified to add the complete adapter sequences required for cluster generation. The finished library was purified from the PCR components. High-throughput sequencing was performed as single-end 75 sequencing using NextSeq 500 (Illumina, Inc., San Diego, CA, USA).

### 4.5. QuantSeq Data Analysis (Quality Control and Assembly)

Sequencing reads were aligned using Bowtie2. The alignment file was used for assembling transcripts and detecting differential expression of genes. Differentially expressed genes were determined based on counts from unique and multiple alignments using coverage in Bedtools. The RC (Read Count) data were performed based on a quantitative normalization method using EdgeR within R using a Bioconductor. Gene classification was performed based on the DAVID (http://david.abcc.ncifcrf.gov/, accessed on 5 July 2020) and Medline (http://www.ncbi.nlm.nih.gov/, accessed on 15 July 2020) databases. All the raw data files used for this experiment were submitted in the NCBI’s BioProject under the accession number of PRJNA807064. The sequencing results for QuantSeq such as the numbers of processed reads, the numbers of mapped reads, and mapping percentages are presented in Supplementary Materials (Table S1).

### 4.6. Functional Annotation and Pathway Analysis of DEGs

Because the QuantSeq data only have 3′-end sequence information, we first searched corresponding gene sequences based on the assembly of sorghum v3.1.1 deposited in the Phytozome database (www.phytozome.org, accessed on 17 August 2020). The differentially expressed genes (DEGs) for the control and salt stress conditions were corrected to a significance level of a fold change ≥4, normalized data ≥4, and *p*-value ≤ 0.05, and the gene ontology (GO) was analyzed using AgriGO (http://systemsbiology.cau.edu.cn/agriGOv2/, accessed on 10 September 2020). For KEGG pathway analysis, DEGs (compared between salt stress conditions and the control) were corrected to a significance level of a fold change ≥3, normalized data ≥6, and *p*-value ≤ 0.05, and we used KEGG Mapper to identify biological pathways.

### 4.7. Cis-Regulatory Elements (CRE) Analysis

After extracting the 1 kb-upstream sequence of predicted genes from the reference genome of sorghum (BTx623), a FASTA file was created using Bedtools. After downloading plant *cis*-regulatory elements data from the New PLACE database (https://www.dna.affrc.go.jp/PLACE/?action=newplace, accessed on 19 August 2020), they were transformed to a FASTA format. The FASTA file of the 1 kb-upstream sequences were searched using BlastN against the CRE FASTA file. The result of BlastN was filtered out only with a similarity of more than 90% and the highest E-value. The DEG data of Nampungchal were matched to the ‘New Place’ *cis*-regulatory elements database to confirm the function of the sorghum gene in relation to the *cis* regulatory elements.

### 4.8. Validation of DEGs by qRT-PCR

The verification of DEG data for five randomly selected genes was performed using real-time PCR with a PP2A gene as the control. The primer was designed using the Pimer3 (v.0.4.0) software. A compact cDNA synthesis kit (Smart Gene, Daejeon, Korea) was used for cDNA synthesis. For qRT-PCR, it was performed using CFX Connect™ Real-Time PCR Detection System (BIO-RAD, Hercules, CA, USA) and SYBR green Q-PCR Master mix (Smart Gene, Daejeon, Korea) according to the manufacturer’s protocols. The qRT-PCR mixture consisted of 2 µL of cDNA, 10 µL of 2× SYBR Green qPCR Master Mix, 2 µL of forward and reverse primers (100 nM each), and 4 µL of ddH_2_O. The PCR reaction for each genes product was carried out with template denaturation and enzyme activation at 95 °C for 10 min, denaturation at 95 °C for 15 s, annealing at 60 °C for 30 s, and extension at 72 °C for 30 s. Data from the PCR runs were analyzed using the average of three biological replications based on Ct values normalized to the average Ct value of the house keeping gene, and the relative expression rate was calculated by the delta Ct (DDCT) method.

## 5. Conclusions

Studies on salt-tolerant plants have been published from various perspectives such as agronomics, physiology, and molecular biology. In this study, we focused on the functional perspectives of salt tolerance mechanisms through physiological and transcriptome analysis. Physiological analysis of the leaves of Nampungchal under high salinity stress showed that it has some avoiding mechanisms not to absorb salt at the beginning of the stress treatment (until 3 DAT); after that period, the response was not very different from other salt-susceptible cultivars. However, this cultivar showed normal growth at 9 DAT despite the similar physiological profiles to other cultivars, indicating that Nampungchal may have an acclimation process during the early stages of the stress treatment by complex genetic interactions. We tried to profile the transcriptome and analyze the CREs under salt stress conditions to find possible clues underlying the tolerant response of this sorghum cultivar. In the current study, we identified 80 genes of Nampungchal connected to *cis*-regulatory elements which are specifically related abiotic stress. The descriptions of these genes were to predict their potential roles under abiotic stress conditions. Among these genes, a total of 55 DEGs (listed in Table 4) are found, which are somehow linked to salt-responsive process in Nampungchal. Those DEGs could be main targets for functional studies in the future. This study will not only provide valuable resources for understanding the genetic control of the salt resistance mechanisms of DEGs to salt stress in sorghum, but will also provide insights for the genetic improvement of salt tolerance genes in the future. Because we already started profiling salt-stress candidate genes and their genetic regulatory background, we are now planning to establish a mapping population by crossing Nampungchal and other sorghum cultivars which are susceptible to salt stress so that we can test molecular markers based on our current study for future salt-improved sorghum cultivars. We hope that this sorghum cultivar will be a decent parental line in our abiotic stress breeding program for sorghum.

## Figures and Tables

**Table 1:** List of genes related to abiotic stress among commonly up-regulated genes in leaves and roots under the salt stress.

Gene	Function
LOC8084733	RING-H2 finger protein ATL3
LOC110430284	RING-H2 finger protein ATL72-like
LOC8065367	chaperone protein dnaJ 20, chloroplastic
LOC8069346	chaperone protein dnaJ 8, chloroplastic
LOC8083217	ethylene-responsive transcription factor ERF109
LOC8086051	ethylene-responsive transcription factor 8
LOC8085844	ethylene-responsive transcription factor 11
LOC8063947	ethylene-responsive transcription factor 4
LOC8086050	ethylene-responsive transcription factor 4
LOC8072153	ethylene-responsive transcription factor 4
LOC8080057	ethylene-responsive transcription factor RAP2-13
LOC8055639	ethylene-responsive transcription factor ERF104
LOC8081902	ethylene-responsive transcription factor RAP2-13
LOC8073540	ethylene-responsive transcription factor ERF060
LOC8082391	ethylene-responsive transcription factor 4
LOC110436144	ethylene-responsive transcription factor 3-like
LOC8054868	dehydration-responsive element-binding protein 1H
LOC8060409	dehydration-responsive element-binding protein 1E
LOC8054869	dehydration-responsive element-binding protein 1A
LOC8054870	dehydration-responsive element-binding protein 1A
LOC8077913	dehydrin DHN1
LOC110431599	protein early responsive to dehydration 15-like
LOC8069003	mitogen-activated protein kinase kinase kinase 2
LOC8069002	mitogen-activated protein kinase kinase kinase 3
LOC8056863	mitogen-activated protein kinase kinase 9
LOC8054176	probable galacturonosyltransferase-like 1
LOC8075735	probable galacturonosyltransferase-like 9
LOC8057368	zinc finger protein ZAT12
LOC8057369	zinc finger protein ZAT5
LOC8086194	zinc finger protein 1
LOC8071266	zinc finger CCCH domain-containing protein 33
LOC8059898	bZIP transcription factor 60
LOC8079022	MADS-box transcription factor 18
LOC8061953	heat stress transcription factor C-2b
LOC8062208	9-*cis*-epoxycarotenoid dioxygenase 1, chloroplastic
LOC8057616	1-aminocyclopropane-1-carboxylate synthase
LOC8059158	transcription factor bHLH13
LOC8080622	receptor-like protein kinase
LOC8078033	cyclin-dependent protein kinase inhibitor EL2
LOC8086215	probable WRKY transcription factor 48
LOC8061067	probable WRKY transcription factor 50
LOC8077628	WRKY transcription factor WRKY71
LOC8077654	transcription factor MYB44
LOC8057074	NAC domain-containing protein 67
LOC8067112	NDR1/HIN1-like protein 6
LOC8071480	K(+) efflux antiporter 3, chloroplastic
LOC8078795	UDP-glycosyltransferase 73C5
LOC8084424	putative cyclic nucleotide-gated ion channel 7
LOC8057765	ankyrin repeat-containing protein NPR4
LOC8086217	UDP-glycosyltransferase 83A1
LOC8078619	IQ domain-containing protein IQM1

**Table 2:** List of genes commonly down-regulated in both leaves and roots grown under salt stress.

Gene	Function
LOC8060217	calmodulin-binding receptor-like cytoplasmic kinase 3
LOC8077055	probable calcium-binding protein CML20
LOC8081289	aquaporin NIP2-2
LOC8076978	gibberellin 20 oxidase 2
LOC8076337	wall-associated receptor kinase 2
LOC8073331	AP2/ERF and B3 domain-containing protein Os01g0141000
LOC8061361	zinc finger protein GIS3
LOC8082590	probable polyamine oxidase 4

**Table 3:** List of *Cis*-regulatory elements (CREs) related to abiotic stress.

CRE	Sequence	Gene	Function
GAREHVAMY1	GGCCGATAACAAACTCCGGCC	barley alpha-amylase gene (Amy 1/6-4)	GARE (gibberellic acid responsive element)
GARE4HVEPB1	GTAACAGAATGCTGG	barley (H.v.) EPB-1 (cysteine proteinase) gene promoter	“GARE-4”; Putative binding site of transcription factor, GAMyB Putative binding site of transcription factor, GAMyB
GREGIONNTPRB1B	TGGCGGCTCTTATCTCACGTGATG	tobacco (N.t.) PRB-1b gene promoter	promoter Binding site of nuclear protein; Contains a G box motif; Contains TAAGAGCCGCC, which is highly conserved in the promoter of ethylene-induced PR genes
HY5AT	TGACACGTGGCA	Arabidopsis bZIP protein HY5	“G box”; HY5 regulates stimulus-induced development of root and hypocotyl
AUXRETGA2GMGH3	TGACGTGGC	putative AUXRE E1 of soybean GH3 promoter	“TGA-box #2”; Strong binding site for proteins in plant nuclear extracts; Called G-box by Liu et al. (1997)
ACIIPVPAL2	CCACCAACCCCC	bean (P.v.) PAL2 promoter	ACII element; Three AC-elements, which are possible Myb protein binding sites, together with a G-box, interact to direct the complex patterns of tissue-specific expression of pAL2 gene
TDBA12NTCHN50	TGACTTTCTGAC	tobacco (N.t.) basic class I chitinase gene (CHN50)	TDBA12 binding site; TDBA12 belongs to WRKY proteins that appear to be unique to plants
SUREAHVISO1	AAAACTAAGAAAGACCGATGGAAAA	barley (H. vulgare) iso1 (encoding isoamylase1) promoter	SURE-a; SUSIBA2 (WRKY transcription factor) binding site; Sugar-responsive element found in barley iso1 promoter
3AF1BOXPSRBCS3	AAATAGATAAATAAAAACATT	pea (P.s.) rbcS-3A gene	3AF1 binding site; 3AF1 site includes a GATA motif
RGATAOS	CAGAAGATA	RTBV promoter	R-GATA (GATA motif binding factor) binding site
BOX1PVCHS15	TAAAAGTTAAAAAC	bean (P.v.) chs15 promoter	Box 1; Resemble the binding site for the GT-1 factor in light-responsive elements
LREBOX2PSRBCS3	TGTGTGGTTAATATG	pea (P.s.) rbcS-3A gene	GT-1 binding; GT-motif
RBENTGA3	TCCAACTTGGA	tobacco (N.t.) GA3 gene promoter	Binding site of RSG (Repression of shoot growth); RSG is a bZIP transcriptional activator
ABRE2HVA1	CCTACGTGGCGG	barley (H.v.) HVA1 gene	ABA responsive element, ABRE2; stress response
ABAREG2	ATGTACGAAGC	sunflower helianthinin	Motif related to ABA regulation
REGION1OSOSEM	CGGCGGCCTCGCCACG	rice (O.s.) Osem gene promoter	ABRE-like sequence; Important for regulation by ABA
ABADESI2	GGACGCGTGGC	wheat histone H3	Synthetic element (hex-3) related to response to ABA and to desiccation
ABRE3OSRAB16	GTACGTGGCGC	rice (O.s.) rab16 and alpha-amylase genes	ABA-responsive element
ABRECE3ZMRAB28	ACGCGCCTCCTC	maize (Z.m.) rab28 gene promoter	ABA responsive element; stress response
ABRECE3HVA1	ACGCGTGTCCTC	barley HVA1 gene	ABRC3 (ABA response complex 3) of HVA1 consists of CE3 and A2; ABA responsive element; stress response
ABREDISTBBNNAPA	GCCACTTGTC	napA gene of Brassica napus (B.n.)	dist B (distal portion of B-box); similarity to ABRE; Required for seed specific expression and ABA responsiveness;
ABRETAEM	GGACACGTGGC	wheat (T.a.) Em gene	ABRE (ABA responsive element)
ABREMOTIFIIIOSRAB16B	GCCGCGTGGC	rice (O.s.) rab16B gene	Motif III; Motif I (S000290) and motif III are both required for ABA responsiveness
ABASEED1	TGTTACGTGCC	carrot Dc3	ABA regulation; seed expression
GBOXRELOSAMY3	CTACGTGGCCA	Amy3D (amylase) promoter of rice (O.s.)	Similar to ABRE; G box-related element
TGA1ANTPR1A	CGTCATCGAGATGACG	tobacco (N.t.) PR1a gene	TGA1a binding site; as-1-like sequence
SRENTTTO1	TGGTAGGTGAGAT	tobacco (N.t.) retrotransposon Tto1	Stress responsive element (SRE) in tobacco (N.t.) retrotransposon Tto1; Involved in responsiveness to tissue culture, wounding, methyl jasmonate
CPRFPCCHS	CCACGTGGCC	parsley (P.c.) light responsive CHS gene promoter	Binding site of CPRF-1, 2, 3, 4(Common Plant Regulatory Factor); CPRF proteins are bZIP class transcription factors
TCA1MOTIF	TCATCTTCTT	TCA-1 (tobacco nuclear protein 1)	TCA-1 (tobacco nuclear protein 1) binding site; Related to salicylic acid-inducible expression of many genes
SGBFGMGMAUX28	TCCACGTGTC	soybean (G.m.) GmAux28 gene promoter	bZIP proteins SGBF-1 and SGBF-2 binding site
VSF1PVGRP18	GCTCCGTTG	French bean (P.v.) grp1.8 gene promoter	VSF-1 binding site; VSF-1 is a tomato bZIP transcription factor
MREATCHS	TCTAACCTACCA	Arabidopsis (A.t.) chalcone synthase (CHS) gene promoter	“MREAtCHS (MRE = Myb Recognition Element)” found in the LRU (light-responsive unit)
ARELIKEGHPGDFR2	AGTTGAATGGGGGTGCA	maize anthocyanin promoter	Sequence highly similar to ARE (anthocyanin regulatory element); Binding site of R2R3-type MYB factor
23BPUASNSCYCB1	TTTATTTACCAAACGGTAACATC	Nicotiana sylvestris (N.s.) CycB1 gene	23 bp UAS (Upstream activating sequence); Contains a 5 bp element identical to the MYB binding core (ACGT)
14BPATERD1	CACTAAATTGTCAC	erd1 in Arabidopsis	“14 bp region” (from −599 to −566) necessary expression of erd1 (early responsive to dehydration) in dehydrated Arabidopsis

**Table 4:** List of Nampungchal DEGs associated with stress-responsive CREs.

Gene	Function	Leaf/Root
LOC8054237	peptidyl-prolyl *cis*-trans isomerase CYP65	down/up
LOC8054689	peptidyl-prolyl *cis*-trans isomerase CYP20-3, chloroplastic	up/down
LOC8057395	peptidyl-prolyl *cis*-trans isomerase CYP22	up/up
LOC8057814	peptidyl-prolyl *cis*-trans isomerase CYP18-2	down/up
LOC8057995	peptidyl-prolyl *cis*-trans isomerase CYP71	down/up
LOC8059503	peptidyl-prolyl *cis*-trans isomerase CYP28, chloroplastic	up/down
LOC8060736	peptidyl-prolyl *cis*-trans isomerase CYP63	up/up
LOC8061594	peptidyl-prolyl *cis*-trans isomerase CYP59	down/up
LOC8062274	peptidyl-prolyl *cis*-trans isomerase CYP19-3	up/up
LOC8064962	peptidyl-prolyl *cis*-trans isomerase CYP19-4	down/down
LOC8064963	peptidyl-prolyl *cis*-trans isomerase CYP19-4	up/down
LOC8064965	peptidyl-prolyl *cis*-trans isomerase CYP20-1	up/up
LOC8068384	peptidyl-prolyl *cis*-trans isomerase CYP18-1	down/down
LOC8070454	peptidyl-prolyl *cis*-trans isomerase CYP26-2, chloroplastic	up/up
LOC8070780	peptidyl-prolyl *cis*-trans isomerase CYP40	down/up
LOC8070908	peptidyl-prolyl *cis*-trans isomerase CYP21-1	up/up
LOC8074358	peptidyl-prolyl *cis*-trans isomerase CYP23	up/up
LOC8078214	peptidyl-prolyl *cis*-trans isomerase CYP57	up/up
LOC8078906	peptidyl-prolyl *cis*-trans isomerase CYP40	up/up
LOC8081760	peptidyl-prolyl *cis*-trans isomerase CYP37, chloroplastic=	up/down
LOC8054768	peptidyl-prolyl *cis*-trans isomerase FKBP15-1	up/up
LOC8059351	peptidyl-prolyl *cis*-trans isomerase FKBP15-1	down/up
LOC8060566	peptidyl-prolyl *cis*-trans isomerase FKBP16-3, chloroplastic	up/up
LOC8061766	peptidyl-prolyl *cis*-trans isomerase FKBP20-1	up/up
LOC8062926	peptidyl-prolyl *cis*-trans isomerase FKBP17-2, chloroplastic	up/down
LOC8069313	peptidyl-prolyl *cis*-trans isomerase FKBP42	up/up
LOC8071644	peptidyl-prolyl *cis*-trans isomerase FKBP16-1, chloroplastic	up/down
LOC8075048	peptidyl-prolyl *cis*-trans isomerase FKBP16-4, chloroplastic	down/down
LOC8075186	peptidyl-prolyl *cis*-trans isomerase FKBP12	up/down
LOC8076111	peptidyl-prolyl *cis*-trans isomerase FKBP17-1, chloroplastic	up/down
LOC8080368	peptidyl-prolyl *cis*-trans isomerase FKBP53	up/up
LOC8080618	peptidyl-prolyl *cis*-trans isomerase FKBP19, chloroplastic	down/up
LOC8080695	peptidyl-prolyl *cis*-trans isomerase FKBP20-2, chloroplastic	up/up
LOC8085209	peptidyl-prolyl *cis*-trans isomerase FKBP18, chloroplastic	up/down
LOC8063484	peptidyl-prolyl *cis*-trans isomerase FKBP53	down/up
LOC8055151	abscisic acid receptor PYR1	down/down
LOC8061804	abscisic acid receptor PYL4	up/down
LOC8065946	abscisic acid receptor PYL8	down/down
LOC8073793	abscisic acid receptor PYL2	up/down
LOC8076724	abscisic acid receptor PYL2	down/up
LOC8078346	abscisic acid receptor PYL8	down/up
LOC8081117	abscisic acid receptor PYL4	down/down
LOC8085416	abscisic acid receptor PYL5	down/down
LOC8064244	abscisic stress-ripening protein 1	up/down
LOC8075331	abscisic stress-ripening protein 1	down/up
LOC8073617	abscisic stress-ripening protein 2	up/down
LOC8075332	abscisic stress-ripening protein 2	up/up
LOC8073983	abscisic stress-ripening protein 3	down/up
LOC8073163	abscisic acid 8′-hydroxylase 1	up/down
LOC8083537	abscisic acid 8′-hydroxylase 2	down/up
LOC8083090	abscisic acid 8′-hydroxylase 3	down/up
LOC8066580	abscisic acid 8′-hydroxylase 4	down/down
LOC8072856	abscisic acid and environmental stress-inducible protein	down/down
LOC8062208	9-*cis*-epoxycarotenoid dioxygenase 1, chloroplastic	up/up
LOC8081132	9-*cis*-epoxycarotenoid dioxygenase 1, chloroplastic	down/down

Leaf/root parts refer to up- and down-regulated genes respectively.

## Data Availability

www.nabic.rda.go.kr (NABIC database, accession#NN-7523-000001, accessed on 15 December 2021).

## Supplementary Materials

The following are available online at https://www.mdpi.com/article/10.3390/plants11070869/s1, Table S1: The sequencing results for QuantSeq.
